# A Systematic Review of Congenital Insensitivity to Pain, a Rare Disease

**DOI:** 10.3390/jpm14060570

**Published:** 2024-05-26

**Authors:** Raquel Rodríguez-Blanque, Laura Maria Nielsen, Beatriz Piqueras-Sola, Juan Carlos Sánchez-García, Celia Cortés-Martín, Andrés Reinoso-Cobo, Jonathan Cortés-Martín

**Affiliations:** 1 San Cecilio University Hospital, 18071 Granada, Spain; rarobladoc@ugr.es; 2 Department of Nursing, Faculty of Health Sciences, University of Granada, 18071 Granada, Spain; celia92cortes@gmail.com (C.C.-M.); jcortesmartin@ugr.es (J.C.-M.); 3 Department of Nursing and Podiatry, Faculty of Health Sciences, University of Malaga—Teatinos, Arquitecto Francisco Peñalosa 3, 29071 Malaga, Spain; lauranielsen2011@hotmail.com (L.M.N.); andreicob@uma.es (A.R.-C.); 4 Virgen de las Nieves, University Hospital, 18014 Granada, Spain; bpiquerassola@gmail.com

**Keywords:** pain insensitivity congenital, rare disease, congenital insensitivity to pain with anhidrosis (CIPA), NTRK1, hereditary sensory and autonomic neuropathies

## Abstract

Introduction: Pain perception, far from being a pathological mechanism, is a crucial protective stimulus to prevent additional injuries. Any disturbance in this complex system poses significant risks to individuals, affecting their quality of life and even their survival. Objective: This review aims to explore congenital insensitivity to pain, an extremely rare genetic disorder with an autosomal recessive pattern that results in the inability to perceive pain. We will focus on the well-known subtype, congenital insensitivity to pain with anhidrosis (CIPA). Our research seeks to update existing knowledge through a comprehensive literature review. Methodology: The review employs a systematic literature review, analyzing various sources and scientific documents, primarily emphasizing CIPA. The review follows the PROSPERO protocol, registered under CRD42023394489. The literature search was performed on the Scopus, PubMed, and Cinahl databases. Results: Our review reveals secondary complications associated with CIPA, such as recurrent bone fractures, temperature insensitivity, self-mutilation, and, occasionally, intellectual disabilities. The limited available information underscores the need for expanding our knowledge. Conclusions: In summary, CIPA, particularly, presents a significant medical challenge with adverse impacts on quality of life. Early diagnosis, education for families and healthcare professionals, and appropriate nursing care are essential for effective management. This review highlights the necessity of further research and awareness to enhance support for those affected.

## 1. Introduction

Congenital insensitivity to pain (CIP) is an extremely rare genetic disease with an autosomal recessive pattern, characterized mainly by a complete inability to perceive pain and temperature stimuli [1,2]. Some reports show that 50% of cases are associated with consanguinity [3]. It was first described by Dearborn in 1932 [4] and classified by Dyck [5] within a group of hereditary sensory and autonomic neuropathies (HSAN), where he distinguished five types based on clinical manifestations and pathological findings [2]. The most described subtype to date is HSAN-IV or congenital insensitivity to pain with anhidrosis (CIPA). The exact prevalence of the disease is currently unknown [6], although some authors estimate an incidence of 1 in 25,000 individuals [2,7]. It is caused by a mutation in the NTRK1 gene [1,8], specifically in the nerve growth factor receptor NGF [9].

CIPA is caused by mutations in the NTRK1 (neurotrophic tyrosine kinase receptor type 1) gene, also known as TRKA. This gene codes for a protein that is a receptor tyrosine kinase, crucial for the development and survival of neurons in the sympathetic and sensory nervous system [8]. Mutations in the NTRK1 gene negatively impact the production and function of the TRKA receptor. This receptor is crucial for neurotrophin signaling, especially nerve growth factor (NGF), which plays a critical role in the survival and function of sensory and sympathetic neurons. Lack of proper signaling through the TRKA receptor prevents these neurons from responding to NGF, leading to apoptosis (programmed cell death) of these cells during development. As a result, there is a deficit of sensory and sympathetic neurons, leading to an inability to perceive pain and temperature, as well as ineffective regulation of sweating and other autonomic functions [7].

CIPA is characterized by the inability to perceive pain and an inability to sweat (anhidrosis). The most common clinical symptoms of the disease are self-inflicted injuries, which result from the inability to feel pain. Patients with CIPA often experience frequent bone fractures and deformities, and many cases also exhibit Charcot joints, which are joints with severe damage due to repeated injury [8].

Moreover, it is important to note that alterations in intelligence levels have been observed in individuals with CIPA. The severity of these intellectual alterations appears to be closely related to the precise location of the genetic mutation responsible for the condition. When the mutation is located at the core of the associated protein, cognitive impairments tend to be more pronounced. In other words, the location of the genetic mutation within the responsible gene seems to be correlated with the degree of cognitive impairment observed in individuals with CIPA [10].

This connection between the location of the genetic mutation and cognitive effects underscores the intricate relationship between the genetic basis of CIPA and its wide-ranging clinical consequences. In summary, CIPA not only affects the ability to perceive pain and sweating but can also lead to intellectual impairments, and the specific genetic mutation’s location plays a significant role in determining the severity of these cognitive effects.

The diagnosis of this condition relies on early recognition of its clinical symptoms, along with genetic sequencing for confirmation. Prognosis hinges on the disease’s severity and the emergence of secondary complications, with a generally reduced life expectancy [6,11].

Currently, treatment options primarily target symptom management, necessitating collaboration among healthcare professionals to address underlying health issues. Education and preventive measures play a significant role in mitigating severe complications [6].

Nursing plays a pivotal role in the care of individuals with this condition, requiring tailored care plans that address each patient’s specific pathologies to enhance their overall quality of life.

Given the limited available information about this disease, there is a growing consensus on the importance of compiling the existing literature into a single document. This compilation underscores the significance of researching such conditions, ultimately leading to early diagnosis and potential therapeutic interventions, thereby minimizing adverse side effects.

This review aims to explore congenital insensitivity to pain, an extremely rare genetic disorder with an autosomal recessive pattern that results in the inability to perceive pain. We will focus on the well-known subtype, congenital insensitivity to pain with anhidrosis (CIPA). Our purpose seeks to update existing knowledge through a comprehensive literature review.

## 2. Methodology

### 2.1. Review Protocol

The methodology used for this report was a systematic review of the scientific literature published on congenital insensitivity to pain, following the Preferred Reporting Items for Systematic reviews and Meta-Analyses (PRISMA) review protocol. PRISMA consists of a checklist of 27 points covering the most representative sections of an original article and the process of developing these guidelines.

This systematic review was conducted following a protocol available at the following website: http://www.crd.york.ac.uk/PROSPERO accessed on 8 February of 2023, with the registration number CRD42023394489.

Recording the review is crucial to ensure transparency and prevent bias. It increases credibility and facilitates collaboration between researchers by making study protocols public. It also improves the quality of research and its visibility in the scientific community.

### 2.2. Eligibility Criteria

As inclusion criterion, we included articles published in the last five years with the aim of ensuring that our bibliography is composed of the most up-to-date sources from a scientific perspective.

### 2.3. Information Sources

The literature search was performed on the Scopus, PubMed, and Cinahl databases. The structured language used was obtained through MeSH terms and Health Sciences Descriptors (DeCS). The descriptors used were “congenital insensitivity to pain” and “rare disease”, with the AND Boolean operator employed.

### 2.4. Search Strategy

The following table (Table 1) shows the search strategy used for this work.

### 2.5. Data Extraction Process

After conducting the search strategy, the found articles were transferred to the Mendeley web application using the Mendeley web importer tool. Subsequently, they were organized into folders based on the database from which they were obtained, and all duplicates were removed.

The found studies consisted of research articles, books, and clinical cases aimed at providing new information about congenital insensitivity to pain syndrome.

In the case of systematic literature reviews, the articles included in these systematic reviews will be studied in order not to duplicate their results by including them independently as research articles.

The reviewers (L.M.N.) and (J.C.-M.) first independently examined the title, abstract, and keywords of each study, applying the inclusion and exclusion criteria. In a second phase, the same procedure was applied to the full text of potentially eligible studies. If the authors had any discussion or doubt about any of the articles, the author (R.R.-B.) would make the decision whether to include it or not.

### 2.6. Data Collection Process and Collected Data

The reviewers extracted the following data from each of the included articles: authors, year of publication, title, main objective, and the most relevant information about the results obtained in each study. The authors screened the articles included in the included systematic reviews to ensure that only single studies would be included in this systematic review. The Section 3 explains in more detail the process of article selection and the information used for the development of this work.

### 2.7. Risk of Bias in Individual Studies

To perform the methodological evaluation of the articles selected for this study, an analysis of the design, methodology, and type of study of each work was conducted to select the most specific methodological evaluation scale for each case.

Out of the 18 articles, 14 were clinical cases, and 5 were literature reviews.

The articles with a case study design were evaluated using the SCED scale (Rating Scale for Single Participants Designs). The SCED scale was constructed with 11 items, of which items 2 to 11 are used to evaluate the methodological quality. Based on the score obtained, they are classified from lower to higher quality, with a maximum methodological quality score of 10 points.

The following table (Table 2) shows the results obtained after conducting the methodological evaluation using the SCED scale.

For the reviews, the Amstar-2 scale (A Measurement Tool to Assess Systematic Reviews) was used for methodological evaluation. Amstar-2 provides a comprehensive assessment of quality, incorporating imperfections that may have arisen due to improper review conduct. Amstar-2 was constructed with 16 domains, each presenting simple response options: “yes” when the criteria are met; “no” if the standard was not achieved or the existing information was insufficient to respond; and “partial yes” in situations where partial adherence to the criteria was observed. Despite not providing an overall score, four levels of confidence emerge: high, moderate, low, and critically low.

Next, the following Table 3 displays the results obtained after conducting the methodological evaluation using the Amstar-2 scale.

## 3. Results

### 3.1. Flow Diagram

**Figure 1:**
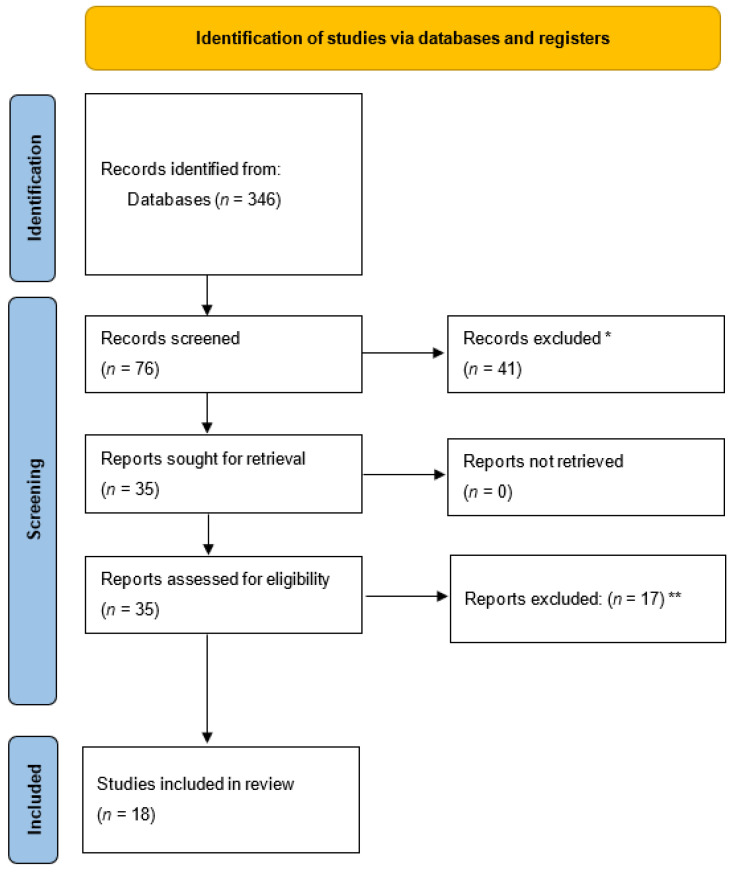
Flow diagram. * Were not in line with the line of research pursued by this systematic review. Articles that have not been published in the last five years and articles that are not available in full text. ** Did not meet the objective proposed by this report.

Below is the flow diagram of the current systematic review (Figure 1).

During the search for articles in the different databases (Scopus, CINAHL, and PubMed), initially, 346 articles were found. These articles were subjected to inclusion and exclusion criteria, so that only articles published in the last 5 years and with full-text availability were considered.

After applying these criteria, 76 articles were considered, which, upon a preliminary reading of titles and abstracts, were further reduced to 35 because they did not fit in with the line of research pursued by this systematic review. These 35 articles were selected for full-text evaluation, and ultimately, this review includes 18 articles. The reason for excluding these last 17 articles after full reading was that they did not meet the objective proposed by this report.

As for the reviews included as outcomes of this work. No articles were observed that were included in the reviews that were also duplicated as individual studies.

### 3.2. Results Table

After selecting and reading the articles, the essential information was obtained from each of them and unified in a summary table to have all the data organized and schematized. In this way, it was easier to visualize the similarities and differences between the articles. The table of results is shown below (Table 4).

Congenital insensitivity to pain (CIPA) represents a rare and intricate medical condition fraught with substantial challenges. Recent investigations have cast a clarifying light on various facets of this disorder, providing valuable insights into aspects encompassing diagnosis, treatment, sensory experiences, and potential cognitive ramifications.

Notably, researchers such as Wang et al. have elucidated the pivotal role played by bone and joint abnormalities in the diagnostic process, particularly in cases correlated with anhidrosis stemming from mutations in the NTRK1 gene [1,10]. While conservative therapeutic approaches, as proposed by Yu et al., have exhibited promise, their suitability may not universally apply, as exemplified by Hartono et al. in a clinical instance where open joint reductions yielded suboptimal results [12,13]. Orthopedic interventions, while efficacious, pose challenges necessitated by obligatory restrictions in movement [18]. In this context, the use of splints, as recommended by Hanatleh et al., presents an alternative therapeutic strategy [8]. Nevertheless, in cases of heightened severity, surgery may become an inescapable recourse, as indicated by Svec et al. [2].

Investigative work by Tsuchihashi et al. pertaining to gustatory and olfactory stimuli divulges that, in general, individuals with CIPA do not experience substantial difficulties with these senses; however, a diminished sensitivity to spicy and acidic tastes is evident [9].

The selection of appropriate anesthesia remains a complex and contentious subject. The utilization of opioids, as scrutinized by Qiu et al., remains a matter of debate, with some scholars proposing its potential superfluousness [17]. Cautionary words have been issued by Takeuchi et al. against the use of remifentanil due to the attendant risk of complications [15]. Similarly, ketamine emerges as contraindicated, as demonstrated by Jiang et al., who have observed an augmented likelihood of aspiration and regurgitation with its application [14]. Jiang et al. and Qiu et al. universally endorse intraoperative precautions, including temperature monitoring and fasting [14,17].

Isolated findings by Elsana et al. have identified novel mutations associated with severe ocular impairments in the context of CIPA [16]. In the specific context of pregnant women afflicted by CIPA, Higeta et al. advocate for cesarean sections over natural childbirth, underlining the paramount importance of safety [19].

A growing awareness is manifesting itself with regard to potential cognitive alterations experienced by CIPA patients. Santoya Montes et al. propose that the impact on higher cognitive functions remains uncertain, while Liu et al. suggest that the severity of intellectual disability may be contingent upon specific amino acid alterations within the NTRK1 gene [11,20].

Authors across the board emphasize the pivotal role of education, particularly for parents, in achieving early diagnoses. Ahmed et al. posit that recurrent febrile episodes and self-mutilation incidents may serve as conspicuous red flags for early detection [3]. Furthermore, the imperative of further research into this enigmatic malady, as underscored by Qiu et al., is advocated to deepen our comprehension of its pathological underpinnings [21]. Additionally, Svec et al., Khurshid et al., and Yu et al. accentuate the indispensable nature of preventive measures to avert potential complications [2,6,13].

## 4. Discussion

The primary objective of this study is to provide an updated understanding of congenital insensitivity to pain. To achieve this, the study’s results, presented in the preceding section, are compared with the existing scientific literature on the same subject.

When elucidating the etiology of this condition, the available information does not present a unanimous consensus. While a majority of studies, such as the research conducted by López Cortés et al., assert that the most well-known mutation responsible for the condition is situated within the NTRK1 gene [1], leading to a reduced survival of pain receptors and sympathetic ganglion neurons [22], authors like Cox et al. contend that variants in the sodium channel subunit SCN9A underlie the etiology of this rare ailment [23].

The SCN9A and SCN11A genes play crucial roles in the transmission of pain signals in CIPA. SCN9A encodes the NaV1.7 sodium channel, present in sensory neurons. This channel amplifies small voltage changes and is essential for initiating and propagating pain signals. Inactivating mutations in SCN9A prevent NaV1.7 from functioning, preventing the generation of action potentials in response to painful stimuli, resulting in complete insensitivity to pain. On the other hand, SCN11A encodes the sodium channel NaV1.9, also present in sensory neurons. NaV1.9 has slow activation and prolonged deactivation, modulating neuronal excitability. Activating mutations in SCN11A cause persistent depolarization of the neuronal membrane, preventing the effective generation and transmission of pain signals. Together, mutations in SCN9A and SCN11A disrupt the proper transmission of pain signals, albeit by different mechanisms, leading to the pain insensitivity characteristic of CIPA [21].

In a related context, Chen et al., in their investigations into the transcriptional regulator PRDM12, propose its indispensable role in pain perception, and suggest that its variants disrupt the development of nociceptive sensory neurons [24].

This diversity of opinions can be attributed to the classification system proposed by Dyck for hereditary autonomic sensory neuropathy. Each of these subtypes is associated with a mutation in distinct genes, and their nomenclature is contingent upon the clinical manifestations they manifest [5].

The subtype linked to anhidrosis (CIPA) is presently the most prevalent and extensively studied variant. This prevalence can be ascribed to the NRTK1 mutation, which is most associated with this specific subtype. In fact, Wang et al. have meticulously documented over 105 distinct mutations at this locus in CIPA patients [10].

This specific subtype is characterized by various secondary issues, including recurrent fractures, self-inflicted injuries, an inability to perceive temperature changes, and, on occasion, cognitive challenges. Svec et al., in their clinical case, have demonstrated that fractures result from the breakdown of protective muscle contractions, leading to the deterioration of the musculoskeletal system and the development of what is known as the Charcot joint [2].

It is pertinent to highlight that while various authors advocate for different approaches to address bone and anesthetic issues, as detailed in the Section 3, there is a consensus in the literature regarding the diagnosis and treatment of this condition [15,17,21].

The diagnosis is typically straightforward. According to Ahmed et al., recurrent febrile episodes, self-mutilation wounds, and bone fractures serve as sufficient causes to establish an early diagnosis of congenital insensitivity to pain [3]. These signs and symptoms are encompassed within the clinical spectrum of this pathology and are primarily induced by the described mutation.

Current treatment approaches primarily aim to alleviate the secondary manifestations of the disease and enhance the patient’s quality of life. In this context, Hanson B et al., in 2019, designed a device to notify individuals of extreme temperature variations in their surroundings [25]. However, no further literature supporting this type of device or proposing new treatment options has been uncovered.

Given the pressing need for effective interventions for this condition, nursing care takes on an even greater significance. To date, there is no scientific literature that directly addresses the role of nursing in managing this syndrome, underscoring the importance of this report.

Nevertheless, nursing professionals play a pivotal role in caring for individuals diagnosed with this disease. Their involvement spans various aspects, including the following:

- Screening and Diagnosis: Nurses may participate in identifying affected individuals, particularly in cases involving children who present with recurring injuries or infections in the absence of pain.
- Education and Counseling: Nurses can educate patients and their families about the nature of the condition, its associated risks, and strategies for daily life management. This encompasses imparting knowledge on injury prevention, health monitoring, and seeking medical care when complications arise.
- Injury Management: Due to the absence of pain sensation, nurses must be well-prepared to manage wounds and injuries. This involves proper wound care techniques, infection prevention, and close follow-up.
- Emotional Support: Patients with congenital insensitivity to pain may encounter emotional and psychological challenges due to their unique condition. Nursing professionals can provide emotional support and assist individuals in coping with the emotional and social aspects of the syndrome.
- Coordination of Care: Nurses can collaborate with other healthcare professionals, such as physicians, therapists, and specialists, to ensure a comprehensive approach to addressing this condition.
- Safety Promotion: Nurses can work with patients and families to implement safety measures in the home and other settings to minimize the risk of undetected injury.

In summary, the relationship between nursing and this syndrome entails delivering specialized care, education, and support to individuals affected by the condition, enabling them to lead safe and healthy lives despite their inability to perceive pain.

This study does have certain limitations. Despite the presence of scientific literature on this condition and its examination in various contexts to establish diagnostic and clinical criteria, there remains insufficient information on the contemporary impact of the disease. Although its prevalence is low, most authors concur that further research on the condition is imperative. A comprehensive approach cannot currently be guaranteed, and significant disparities persist in the literature concerning its various subtypes.

Emerging from this study are new avenues for future research, which underscore the pressing need to develop a standardized nursing care plan that serves as a reference point. This approach aims to equip both family members and healthcare professionals with knowledge and skills for managing this disorder. Nursing diagnoses, encompassing areas like health education and self-harm risk assessment, among others, are essential to mitigate the side effects associated with this condition.

Furthermore, it would be beneficial to establish specific training programs, such as courses or seminars, for nursing professionals and other healthcare practitioners to gain an in-depth understanding of this rare pathology.

## 5. Conclusions

Commencing with an initial inquiry into the adequacy of the literature and comprehension regarding congenital insensitivity to pain, our investigation has uncovered that, upon meticulous scrutiny of the most recent data available up to the present day, a glaring inadequacy in our knowledge base becomes evident.

It is important for us to underscore the necessity of imparting knowledge, not only to healthcare professionals but also to families, enabling the early detection of this condition and, in doing so, curbing the onset of intricate secondary complications. Of particular significance is the emphasis on preventing self-inflicted injuries.

In conclusion, congenital insensitivity to pain is a condition that unequivocally warrants further research to augment the overall quality of life for individuals afflicted by this rare disorder.

## Figures and Tables

**Table 1:** Search strategy.

Database	Search Strategy
SCOPUS	(TITLE-ABS-KEY (congenital AND insensitivity AND to AND pain) AND TITLE-ABS-KEY (rare AND disease))
PUBMED	((“pain insensitivity, congenital”[MeSH Terms] OR (“pain”[All Fields] AND “insensitivity”[All Fields] AND “congenital”[All Fields]) OR “congenital pain insensitivity”[All Fields] OR (“congenital”[All Fields] AND “insensitity”[AllFields] AND “pain”[All Fields]) OR “congenital insensitivity to pain”[AllFields]) AND (“rare diseases”[MeSH Terms] OR (“rare”[All Fields] AND “diseases”[All Fields]) OR “rare diseases”[All Fields] OR (“rare”[All Fields] AND “disease”[All Fields]) OR “rare disease”[All Fields])) AND ((y_5[Filter]) AND (ffrft[Filter]))
CINAHL	Congenital insensitivity to pain AND rare disease

**Table 2:** Methodological evaluation according to SCED.

Author	Article	Numerical Score
López-Cortés et al. [1]	Clinical, genomics and networking analyses of a high-altitude native American Ecuadorian patient with congenital insensitivity to pain with anhidrosis: a case report	9: High quality.
Svec et al. [2]	Congenital insensitivity to pain in one family	9: High quality.
Ahmed et al. [3]	A case report of two bahraini siblings presenting with different rare neurogenetic disorders: congenital insensitivity to pain with anhidrosis and rigid spine muscular dystrophy	7: High quality.
Khurshid et al. [6]	Not another case of juvenile idiopathic arthritis: congenital insensitivity to pain presenting with joint problems.	8: High quality.
Hanatleh et al. [8]	A 5-Year-Old palestinian bedouin girl with repeated self-induced injuries to the digits, a diagnosis of congenital insensitivity to pain, and anhidrosis	8: High quality.
Tsuchihashi et al. [9]	Perception of pungent, gustatory and olfactory stimuli in patients with congenital insensitivity to pain with anhidrosis.	10: High quality.
Hartono et al. [12]	Catastrophic results due to unrecognizing of congenital insensitivity to pain with anhidrosis in children with multiple long bones fractures: A case report of 27 years follow-up of two siblings	9: High quality.
Yu et al. [13]	Conservative treatment or surgical treatment: a case report and literature review of multiple fractures of the lower extremities in a child with insensitivity to pain	8: High quality.
Jiang et al. [14]	A case report: anesthetic management for open-heart surgery in a child with congenital insensitivity to pain with anhidrosis	8: High quality.
Takeuchi et al. [15]	Anesthetic management of a patient with congenital insensitivity to pain with anhidrosis by coadministration of remifentanil	8: High quality.
Elsana et al. [16]	Ocular manifestations among patients with congenital insensitivity to pain due to variants in PRDM12 and SCN9A genes	9: High quality.
Qiu et al. [17]	Anesthetic management of children with congenital insensitivity to pain with anhidrosis	7: High quality.
Al Kaissi et al. [18].	Unilateral lytic changes over the weight-bearing joint causing severe destruction of ankle joint (atypical Charcot joint) in a girl with congenital insensitivity to pain without anhidrosis (hereditary sensory and autonomic neuropathy type V): case report and literature review	8: High quality.
Higeta et al. [19]	Pregnancy in hereditary sensory and autonomic neuropathy type V: a case report and literature review	7: High quality.

**Table 3:** Methodological evaluation according to Amstar-2.

Author	Article	Numerical Score
Liu et al. [20]	Phenotypic heterogeneity of intellectual disability in patients with congenital insensitivity to pain with anhidrosis: A case report and literature review	7
Santoya Montes et al. [11]	Clinical manifestations of congenital Insensitivity to pain with anhidrosis	6
Wang et al. [10]	Postoperative redislocation of the hip in a patient with congenital insensitivity to pain with anhidrosis: A case report and review of literature	6
Zhao et al. [21]	Trends in congenital insensitivity to pain with anhidrosis: a bibliometric analysis from 2000 to 2021	7

**Table 4:** Results table.

Author	Year	Title	Aims	Results
López-Cortés et al. [1]	2020	Clinical, genomics and networking analyses of a high-altitude native American Ecuadorian patient with congenital insensitivity to pain with anhidrosis: a case report	Demonstrate the genetic mutation causing CIPA.	CIPA is caused by a mutation in NTRK1.
Svec et al. [2]	2018	Congenital insensitivity to pain in one family	Presenting a clinical case about two siblings with congenital insensitivity to pain.	Conservative treatment is viable for mild injuries, but in severe cases, surgery cannot be avoided. Prevention of self-harm is necessary.
Ahmed et al. [3]	2022	A Case Report of Two Bahraini Siblings Presenting with Different Rare Neurogenetic Disorders: Congenital Insensitivity to Pain with Anhidrosis and Rigid Spine Muscular Dystrophy	Report a clinical case of two siblings with neurogenetic diseases.	An early diagnosis is required, based on, among others, recurrent febrile episodes and episodes of self-mutilation.
Khurshid et al. [6]	2021	Not Another Case Of Juvenile Idiopathic Arthritis: Congenital Insensitivity To Pain Presenting With Joint Problems.	Presenting a clinical case of a girl with joint issues and swelling.	As the current treatment is symptomatic, it is necessary to emphasize prevention and education about self-harm to avoid severe complications.
Hanatleh et al. [8]	2021	A 5-Year-Old Palestinian Bedouin Girl with Repeated Self-Induced Injuries to the Digits, a Diagnosis of Congenital Insensitivity to Pain, and Anhidrosis	Demonstrate the importance of diagnosis and treatment in patients with congenital insensitivity to pain. clinical case of a 5-year-old girl with self-inflicted injuries.	Immobilization with plaster casts in the treatment of orthopedic complications is important.
Tsuchihashi et al. [9]	2021	Perception of pungent, gustatory and olfactory stimuli in patients with congenital insensitivity to pain with anhidrosis	Clarify the ability of patients with congenital insensitivity to pain with anhidrosis (CIPA) to perceive spicy, gustatory, and olfactory stimuli.	Gustatory and olfactory stimuli are not altered. There is a higher threshold for detecting capsaicin (spiciness) and sour taste.
Wang et al. [10]	2018	Postoperative redislocation of the hip in a patient with congenital insensitivity to pain with anhidrosis: A case report and review of literature	Describing a case of postoperative hip relocation in a patient with CIPA.	The diagnosis of patients with mild traumas resulting in fractures, without sensitivity or sweating, corresponds to CIPA.
Santoya Montes et al. [11]	2021	Clinical manifestations of congenital Insensitivity to pain with anhidrosis	Review the clinical symptomatology and neurocognitive alterations reported in 145 cases of patients with CIPA from 2000 to 2017.	It has cognitive implications. The level of impact of the disease on the behavior and higher functions of the affected patients is unknown.
Hartono et al. [12]	2020	Catastrophic results due to unrecognizing of congenital insensitivity to pain with anhidrosis in children with multiple long bones fractures: A case report of 27 years follow-up of two siblings	Describing a follow-up of two brothers over 27 years with multiple fractures.	Fractures in joints treated with open reduction have disastrous outcomes.
Yu et al. [13]	2020	Conservative Treatment or Surgical Treatment: A Case Report and Literature Review of Multiple Fractures of the Lower Extremities in a Child with Insensitivity to Pain	Explain the therapeutic approach of a patient with congenital insensitivity to pain.	The conservative, surgical, and problem-based treatment is currently yielding the best results. Additionally, it is essential for parents to observe their children’s behavior to achieve an early diagnosis.
Jiang et al. [14]	2022	A case report: Anesthetic management for open-heart surgery in a child with congenital insensitivity to pain with anhidrosis	Explain the anesthetic approach for a patient with CIPA.	Ketamine should not be used because it increases nausea, vomiting, and the risk of aspiration and regurgitation. Fasting time should be extended for patients with CIPA
Takeuchi et al. [15]	2018	Anesthetic management of a patient with congenital insensitivity to pain with anhidrosis by coadministration of remifentanil	Demonstrate the effectiveness of intraoperative remifentanil.	Remifentanil is beneficial in patients with CIPA. However, it should be used with caution because low doses cause hyperalgesia, and high doses cause chills.
Elsana et al. [16]	2022	Ocular manifestations among patients with congenital insensitivity to pain due to variants in PRDM12 and SCN9A genes	Explain ocular manifestations in patients with congenital insensitivity to pain	A new variant of PRDM12 causes more severe ocular impairments than those with the SCN9A variant.
Qiu et al. [17]	2020	Anesthetic management of children with congenital insensitivity to pain with anhidrosis	Explain the anesthetic approach in congenital insensitivity to pain.	Opioids are not necessary. It is of special importance to control the patient’s temperature.
Al Kaissi et al. [18]	2019	Unilateral lytic changes over the weight-bearing joint causing severe destruction of ankle joint (atypical Charcot joint) in a girl with congenital insensitivity to pain without anhidrosis (hereditary sensory and autonomic neuropathy type V): Case report and literature review	Describing the case of a 13-year-old boy with bone issues and fractures.	Orthopedic treatment in these patients is complicated due to the lack of pain-related movement restriction. Interestingly, in this case, there was a successful outcome.
Higeta et al. [19]	2022	Pregnancy in hereditary sensory and autonomic neuropathy type V: A case report and literature review	Describe the treatment of pregnancy and childbirth for a woman with HSAV-V (Herpes Simplex Virus-Varicella-Zoster).	Cesarean section appears favorable over vaginal delivery in women.
Liu et al. [20]	2018	Phenotypic heterogeneity of intellectual disability in patients with congenital insensitivity to pain with anhidrosis: A case report and literature review	Determining the association between mutations and intellectual disability in patients with CIPA.	Mutations in critical amino acids of the NTRK1 protein are likely to cause severe symptoms, including intellectual disability. Peripheral mutations do not influence important domains and therefore lead to mild symptoms without disability.
Zhao et al. [21]	2022	Trends in Congenital Insensitivity to Pain with Anhidrosis: A Bibliometric Analysis from 2000 to 2021	Describing contributions over the years to CIPA.	More research and cooperation are needed to study the pathological mechanisms of the disease.

## Data Availability

Data regarding the study are available upon request to the corresponding author.
